# Remote Ischemia Postconditioning Mitigates Hippocampal Neuron Impairment by Modulating Cav1.2-CaMKIIα-Aromatase Signaling After Global Cerebral Ischemia in Ovariectomized Rats

**DOI:** 10.1007/s12035-024-03930-1

**Published:** 2024-02-06

**Authors:** Lu Wang, Fujia Gao, Lingling Chen, Wuxiang Sun, Huiyu Liu, Wei Yang, Xin Zhang, Jing Bai, Ruimin Wang

**Affiliations:** 1 Neurobiology Institute, School of Public Health, North China University of Science and Technology, Tangshan, 063210 Hebei China; 2 Dementia and Dyscognitive Key Lab., North China University of Science and Technology, International Science & Technology Cooperation Base of Geriatric Medicine of China, 21 Bohai Road, Caofeidian Xincheng, Tangshan, 063210 Hebei China; 3 Hebei Key Laboratory of Occupational Health and Safety for Coal Industry, Tangshan, Hebei China

**Keywords:** Global cerebral ischemia, Remote ischemia postconditioning, Brain-derived estrogen, CaMKIIα, Neuroprotection

## Abstract

Brain-derived estrogen (BDE2) is gaining attention as an endogenous neurotransmitter. Recent research has revealed that selectively removing the aromatase gene, the pivotal enzyme responsible for BDE2 synthesis, in forebrain neurons or astrocytes can lead to synaptic loss and cognitive impairment. It is worth noting that remote ischemia post-conditioning (RIP), a non-invasive technique, has been shown to activate natural protective mechanisms against severe ischemic events. The aim of our study was to investigate whether RIP triggers aromatase-BDE2 signaling, shedding light on its neuroprotective mechanisms after global cerebral ischemia (GCI) in ovariectomized rats. Our findings are as follows: (1) RIP was effective in mitigating ischemic damage in hippocampal CA1 neurons and improved cognitive function after GCI. This was partially due to increased Aro-BDE2 signaling in CA1 neurons. (2) RIP intervention efficiently enhanced pro-survival kinase pathways, such as AKT, ERK1/2, CREB, and suppressed CaMKIIα signaling in CA1 astrocytes induced by GCI. Remarkably, inhibiting CaMKIIα activity led to elevated Aro-BDE2 levels and replicated the benefits of RIP. (3) We also identified the positive mediation of Cav1.2, an LVGCC calcium channel, on CaMKIIα-Aro/BDE2 pathway response to RIP intervention. (4) Significantly, either RIP or CaMKIIα inhibition was found to alleviate reactive astrogliosis, which was accompanied by increased pro-survival A2-astrocyte protein S100A10 and decreased pro-death A1-astrocyte marker C3 levels. In summary, our study provides compelling evidence that Aro-BDE2 signaling is a critical target for the reparative effects of RIP following ischemic insult. This effect may be mediated through the CaV1.2-CaMKIIα signaling pathway, in collaboration with astrocyte-neuron interactions, thereby maintaining calcium homeostasis in the neuronal microenvironment and reducing neuronal damage after ischemia.

## Introduction

Global cerebral ischemia (GCI) can be caused by various factors, including cardiac arrest, asphyxia, hypotensive shock, among others, in both humans and animals. This codition leads to the selective death of the hippocampal CA1 neurons and cognitive dysfunction. Currently, there are no clinically effective measures except for mild hypothermia treatment. However, meta-analysis shows that patients have not experienced obvious neurological recovery after gentle hypothermia treatment, highlighting the urgent need to explore new treatment methods.

The impact of 17β-estradiol (E2) on the central nervous system and hormone replacement therapy (HRT) has sparked global interest in neuroprotection. Research has revealed that young women have a significantly lower incidence of cerebrovascular disease than men of the same age due to the protective effects of E2. However, postmenopausal women are at a higher risk and experience worse outcomes, such as a 1.8 times higher incidence of Alzheimer’s disease in 65-year-old women compared to their male counterparts [1]. These findings emphasize the critical role of E2 against neurological diseases, including acute stroke and neurodegenerative diseases. However, it is worth noting that the Women’s Health Initiative discovered that HRT unexpectedly increased the risk of stroke in a large-scale clinical trial on over 64-year-old women, halting its clinical application [2, 3]. Consequently, researchers including ourselves have proposed hypotheses such as the “critical period,” [4] “estrogen receptor degradation,” [5] and “healthy cell bias of E2,” [6, 7] to explain the failure of HRT. Essentially, the beneficial effects of E2 occur during perimenopause when estrogen receptors have not yet decreased and neurons are still healthy. Therefore, exogenous E2 treatment prevents cerebrovascular diseases more effectively than repairing them after brain injury. Hence, repairing damaged neurons is crucial in the current clinical treatment of ischemic encephalopathy.

Apart from gonadal-derived E2, it is worth noting that the brain can convert testosterone into E2 (brain-derived E2, BDE2) through aromatase, an key enzyme that is widely distributed in the hippocampus, cortex, hypothalamus, and other brain regions [8, 9]. Like exogenous E2, locally synthesized E2 in the brain has beneficial effects, such as elevated neurogenesis, synapsis, sd, and cognitive promotion [10]. Interestingly, the level of BDE2 remains almost six times higher than circulating E2 in postmenopausal women when serum E2 significantly declines due to ovary dysfunction [11]. Moreover, the autopsy reports of older women with Alzheimer’s disease have show that aromatase levels are significantly lower than those of the control group of the same age [12]. Our recent research demonstrated that BDE2 is crucial to maintaining brain function during aging [13]. It is worth noting that BDE2 synthesis is a more active response to damage stimuli, such as ischemia. In stroke and GCI animal models, expression and activity of aromatase significantly enhance in activated astrocytes, while central knockdown/knockout or inhibitor of aromatase deteriorates the brain injury [8, 14–16]. Recently, post-mortem analysis on 0- to 9-year-old children showed that experiencing neuro-inflammation could upregulate the mRNA level of aromatase and promote BDE2 synthesis [17]. Therefore, it is still largely unclear how to induce BDE2 production in brain injury repair and how aromatase mediates BDE2 biosynthesis.

Remote ischemic postconditioning (RIP) is a novel therapeutic approach that involves the application of one or more non-lethal cycles of ischemia and reperfusion to a rat’s hindlimbs following severe ischemic injury. A significant body of research has demonstrated that RIP is capable of eliciting self-defense mechanisms against inflammation, oxidative stress, and promoting blood-brain barrier function and cognitive function in experimental models of stroke [18–25]. It is even more exciting that these protective effects have also been observed in humans as well [26, 27]. Moreover, several studies have reported that RIP can mediate astrocytic reactivity, thereby safeguarding neurological function [28, 29]. This therapeutic approach holds great promise for promoting neuroprotection in ischemic injury.

Given the above findings, we sought to investigate whether RIP could induce BDE2 in the ischemic brain and, if so, the possible mechanisms involved. In our study, we utilized the aromatase inhibitor letrozole to confirm that RIP could induce aromatase-BDE2 signaling in response to GCI insult, potentially via the CaV1.2-CaMKII α pathway. Our results reveal a novel role of RIP in preventing neuronal mitigation in OVX-rats after GCI and provide compelling evidence for its potential as a viable approach for repairing cerebral ischemia-reperfusion injury.

## Materials and Methods

### Antibodies and Reagents

The following antibodies were utilized in this study: Aromatase (Arigo, 22848), Estrodiol (BioGennex Cat# ARO380913), NenN (MilRIPore, NG1857584), GFAP (Abcam, ab53554), S100A10 (ABclonal, A1987), C3 (ABclonal, A6897), Cav1.2 (Abcam, ab84814), p-CaMKIIα (Cell-signaling, 3361S), CaMKIIα (Cell-signaling, 50049S), p-AKT (Cell-signaling, 4060S), AKT (Proteintech, 10176-2-AP), p-ERK1/2 (Cell-signaling, 4370S), ERK1/2 (Abcam, ab50011), p-CREB (Cell-signaling, 9198S), CREB (Cell-signaling, 9197X), BDNF (Abcam, ab108319), PSD95 (Abcam, ab18258), Spinophilin (Cell-signaling, 14136S), α-Tubulin (Abcam, ab7291), and GAPDH (Santa Cruz, SC-32233). Letrozole (L6545) was obtained from Sigma-Aldrich.

Moreover, the tatCN21 peptide (GRKKRRQRRR-NH2-KRPPKLGQIGRSKRVVIEDDR-COOH) peptide was synthesized by 21^st^ Century Biochemicals according to the previous report [30, 31]. The tatCN21 peptide comprises 21 amino acids linked to the human immunodeficiency virus (HIV)-tat peptide, which facilitates entry into the cell where the CN21 sequence binds to the catalytic domain of activated CaMKIIα and inhibits its autonomous activity.

### Animals and GCI Model

Female Sprague Dawley rats aged 3 months (weighing 250-300 g) were purchased from Beijing HFK Biotechnology Co., Ltd. (SCXK [Jing] 2020-0004). The rats were group-housed, with a maximum of four rats per cage, in environmentally controlled conditions (22-24°C) with a 12:12/h light/dark cycle. They were provided with food and water ad libitum.

To induce an estrogen-deprivation model, the rats underwent bilateral oophorectomy under isoflurane anesthesia and were then randomly assigned to five groups: sham group (sham, sham + RIP), ischemia/reperfusion group (IR, 3h, 3d, and 7d), RIP intervention group (IR + RIP, 3h, 3d, and 7d), letrozole-treated group (IR + RIP + Let, 7d), and TatCN21-administrated group (IR + TatCN21, 7d). All animal experiments were approved by the Institutional Animal Care and Use Committee of North China University of Science and Technology (Protocol # Ref. 2016047) and were performed in accordance with the guidelines for the Care and Use of Laboratory Animals, National Natural Science Foundation of China.

Global cerebral ischemia (GCI) was conducted one week after OVX using four-vessel occlusion, as previously described [32]. Briefly, both vertebral arteries of the rats were electrocauterized through the alar foramen of the first cervical vertebra. The following day, both common carotid arteries were exposed, and the incision was closed with a suture. After 24 h, the rats were lightly anesthetized to re-expose and occlude the CAA for 12 min using aneurysm cRIPs, followed by reperfusion for 3h, 3d, and 7d, respectively. Rats that lost their righting reflex within 30 s and whose pupils were dilated and unresponsive to light during ischemia were considered successful and selected for the experiments. The resumption of carotid artery blood flow was visually confirmed by releasing the cRIPs. Rectal temperature was maintained at approximately 36.5–37.5 °C during the procedure using an incubator. Sham-operated animals underwent the same procedure as ischemic animals, except that the CCA was not clamped.

### RIP Intervention and Drug Administration

To perform RIP intervention, the proximal femoral artery was isolated, and blood flow was blocked for 5 min (ischemia), followed by a 5-min release (reperfusion) for 5 cycles (40 min in total). The successful induction of limb ischemia was confirmed by the disappearance of the pulse, hypothermia, and skin cyanosis in the limb. In the IR + RIP group, RIP was carried out at the end of the 12-min ischemia period.

Letrozole, an aromatase inhibitor (30 μg/d, a total of 4d), or CaMKIIα specific inhibitor, tatCN21 peptide (50 μg/rat) was administered bilaterally intracerebroventricularly (*icv*) at 3 h after GCI. The vehicle-treatment group received the same volume of corn oil with 1% DMSO for letrozole or negative control of tatCN21 at the same time points as letrozole or tatCN21 administration.

### Immunofluorescence Staining and Confocal Microscopy

Immunofluorescence staining was carried out as previously described [33]. Briefly, the rats were sacrificed at indicated time points under deep anesthesia, followed by cardiac perfusion using 0.9% saline. The whole brain was rapidly removed; cerebellum and brainstem were cut and discarded. The brain was separated into both hemispheres, one was rapidly dissected to obtain hippocampus tissue frozen in liquid for western blot analysis. For immunofluorescence staining, the other hemisphere was dehydrated entirely with 30% sucrose after a post-fixed overnight at 4°C with 4% paraformaldehyde, and then frozen sections (25 μm each) were made in series in the coronal plane of the dorsal hippocampus (∼2.5–4.5 mm posterior from Bregma, ∼100 sections per brain). The sections were washed in 0.1 M PBS for 30 min, permeabilized with 0.4% Triton X-100-PBS for 2 h, and incubated with 10% normal donkey serum for 1 h at room temperature. The sections were incubated overnight at 4°C with the following primary antibodies: E2 (working solution), NeuN (1:300), Aromatase (1:200), GFAP (1:1000), S100A10 (1:200), C3 (1:100), and CaMKIIα (1:100). After washing three times for 30 min in 0.4% Triton X-100-PBS, the sections were incubated with Highly Cross-Adsorbed Alexa Fluro IgG second antibodies (Thermo Fisher Scientific, donkey anti-mouse 488 nm/594 nm, donkey anti-rabbit 488 nm/594 nm, donkey anti-goat 594 nm) for 1 h at room temperature. Following three final washes for 10 min each, sections were mounted and coversRIPped in Vectashield mounting medium with DAPI (H-1200; Vector Laboratories, Inc., CA, USA). All confocal images were captured on an FV1000 confocal laser microscope (Olympus) and digital imaging software (FV10-ASW 1.5 Viewer). For quantitative analysis, the number of surviving neurons per 250 μm length of medial CA1 pyramidal cell layer was counted bilaterally in four to six representative sections per animal. Furthermore, the fluorescence intensity of the targeting protein was normalized as the percent change compared to the control group as indicated in the figures. If necessary, colocalization was analyzed using Fiji software (version 1.52q).

### Preparation of Protein Samples and Western Blot Analysis

Preparation of protein samples and western blot analysis were performed as previously described [34]. In brief, the hippocampus tissues were homogenized in ice-cold tissue protein extraction buffer consisting of (in mM) the following: 50 HEPES PH 7.4, 150 NaCl, 1 β-glycerophosphate, 3 DTT, 2 Na_3_VO_3_, 1 EDTA, 1 EGTA, 1 NaF, 1 phenylmethylsulfonyl fluoride (PMSF), 1% Triton X-100 and Protease & Phosphatase Inhibitors Cocktail (#1861280, Thermo Scientific. USA). The homogenates were centrifuged at 15 000 g for 30 min at 4 °C, and then, the supernatants were collected and stored at −80°C until use. The protein concentrations were determined using an enhanced BCA protein assay kit (P0009, Beyotime Institute of Biotechnology, China) with bovine serum albumin (BSA) as the standard. Protein samples were heated at 100°C for 5 min with a loading buffer containing 0.125 M Tris-HCL (PH 6.8), 20% glycerol, 4% SDS, 10% mercaptoethanol, and 0.002% bromphenol blue, then separated by sodium dodecyl sulfate-polyacrylamide gel electrophoresis (SDS-PAGE) of 5% or 10% gels (20 μg protein per lane). Then, the proteins were transferred onto PVDF membranes using a wet transfer system at 220 mA. Blotting membranes were incubated with 3% BSA, 0.2% Tween 20 in TBST for 1 h at room temperature and probed using the antibodies overnight at 4°C. The following antibodies were used for the current study: Aromatase (1:500), CaV1.2 (1:1000), p-CaMKIIα (1:1000), CaMKIIα (1:1000), p-AKT (1:1000), AKT (1:1000), p-ERK1/2 (1:1000), ERK1/2 (1:250), p-CREB (sc-7981), CREB (sc-45), BDNF (sc-10748), PSD95 (1:500), GFAP (1:500), S100A10 (1:200), spinophilin (1:500) and GAPDH (1:1000), α-Tubulin (1:3000). After washing with TBST for 3 × 10 min, the membranes were probed with corresponding horseradish catalase (HRP) conjugated second antibodies at room temperature for 1.5 h. followed by washing, the membranes were soaked in ECL solution and scanned by a chemiluminescence developer. The intensities of the bands were quantified using ImageJ analysis software (version 1.30 v; Wayne Rasband, National Institutes of Health, Bethesda, MD). The band densities for the indicated proteins were corrected for variations in loading and normalized relative to GAPDH or Tubulin. A mean ± SEM was calculated from the data from all of the animals for graphical presentation and statistical comparison.

### Morris Water Maze Test

Morris water maze test was performed on days 4–7 after GCI as previously described [35]. In brief, the test consists of two parts, latency trial and probe trial, representatively testing spatial learning and memory function. A circular pool filled with water (1.2 m in diameter, 35 cm in height) containing a platform concealed below the surface (2.0 cm) was used and was equally divided into four quadrants. During the latency trial, adaptive training was first performed in the morning. The rats were randomly placed in one of the quadrants facing the pool wall and allowed to swim for a maximum time of 90 s until they discovered the fixed platform. If the rat was unable to find the platform within 90 s, it was gently guided to the platform and allowed to rest on the platform for 20 s. Six hours after the adaptive training, formal testing was conducted. The training procedure was repeated four times a day, starting from different quadrants with a 2 min intertrial interval for 3 consecutive days. The escape latency, time to reach the hidden platform and swimming speed were recorded. The day 4 of the Morris water maze test, a 90 s memory probe trial was performed by removing the hidden platform from the pool. The rats were placed in the pool in the exact random start location, and the time spent in the quadrant that previously contained the platform was used to evaluate the level of spatial reference memory. All behavioral tracks from the trials were recorded and analyzed using any maze software.

### Statistical Analysis

SigmaStat 3.5 software was used to analyze all data. Data are presented as mean ± SEM. A T-test was used for comparison between the two groups. One-way ANOVA tests were conducted to compare three or more groups. When the ANOVA test was found to be significant, the Student-Newman-Keuls (S-N-K) was conducted to make pair-wise comparisons to determine the significance between the two groups. For the latency trial of the Morris water maze, the time taken by the rat to find an underwater platform from four quadrants was averaged as latency time and conducted RM one-way ANOVA followed by S-N-K analysis. Statistical significance was accepted at the 95% confidence level (*P* < 0.05). *, **, and *** were presented *P*<0.05, *P*<0.01, and *P*<0.001, respectively.

## Results

### Mediation of RIP Intervention on Aromatase-Estrogen Signaling in the Hippocampal CA1 Region of OVX-Rats Following GCI

**Fig. 1:**
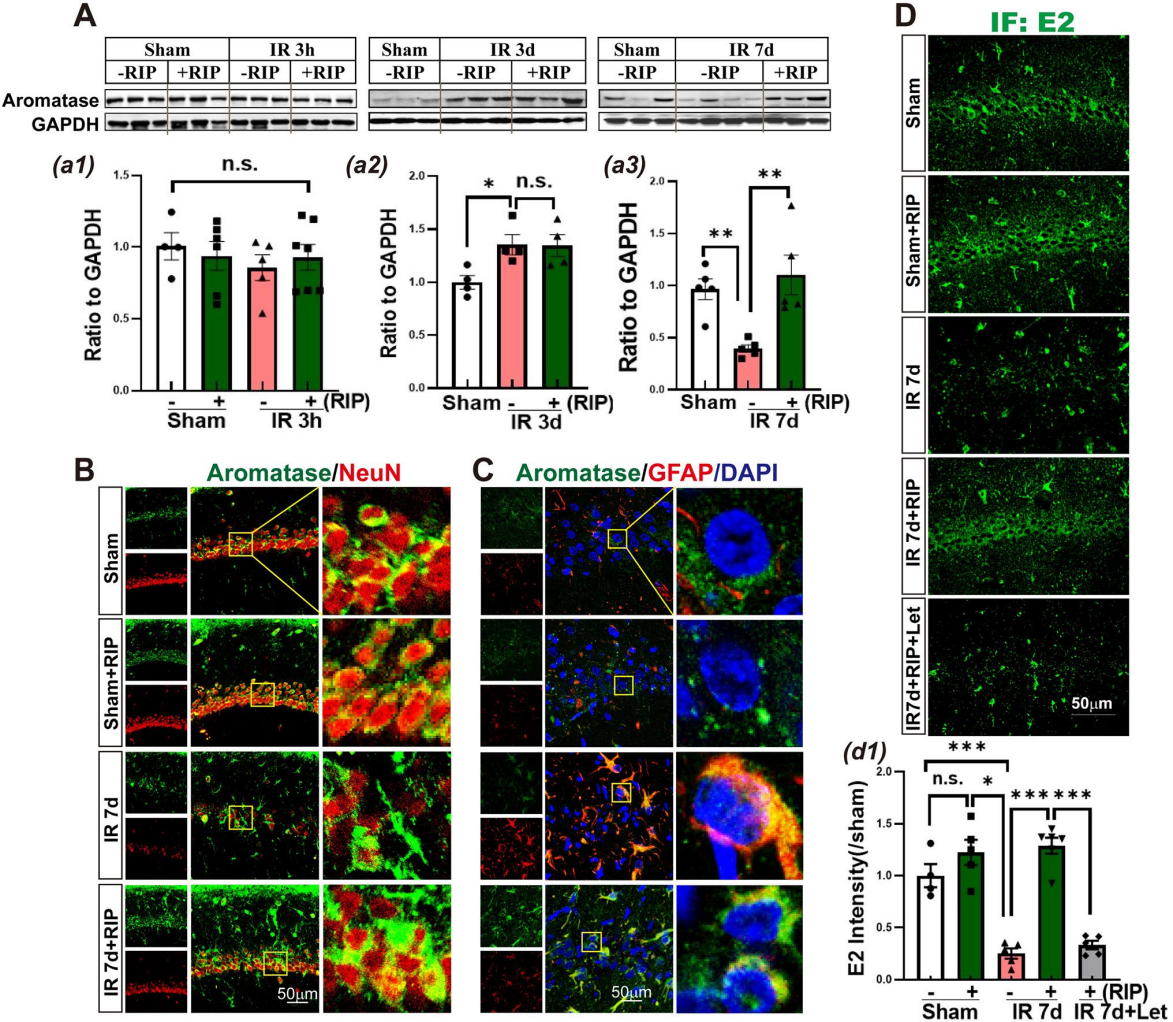
The effects of RIP on aromatase expression and E2 level in the hippocampal CA1 region following GCI. **A** Western blot analysis showed aromatase expression in the indicated time points with or without RIP intervention. Values are means SEM of determinations from each group (a1–a3). GAPHD was used as the loading control. *P<0.05, **P<0.01, n.s. means no significance, *n*=4–7. Representative photographs of double immunofluorescence staining for aromatase (green) with NeuN (red) (**B**), and for aromatase (green) with GFAP (red) (**C**) in the indicated groups. DAPI counterstaining was used to visualizethe nucleus of cells. **D** Representative photographs of E2 immunofluorescence staining in the indicated group. Scale bar 50 μm, magnification ×40. IR: ischemia-reperfusion; RIP: remote ischemic postconditioning; Let: letrozole

We first investigated the time course of aromatase protein expression in the hippocampal CA1 region with and without RIP intervention following GCI. Western blot analysis showed that RIP had no significant effect on aromatase protein expression levels in sham and IR 3h animals. Additionally, IR 3h did not induce a statistical difference in aromatase protein level compared to the Sham group (Fig. 1A, a1). However, at IR 3d, aromatase protein level was significantly enhanced compared with the sham group, but there was no significant difference in aromatase expression between the RIP-treated and non-RIP rats (Fig. 1A, a2). Interestingly, IR 7d induced a significant decrease in aromatase expression compared with the sham group, while RIP intervention reversed this change, resulting in a robust increase in aromatase protein expression in the hippocampal CA1 region (Fig. 1A, a3).

We also conducted double immunofluorescence staining for aromatase with NeuN and GFAP to observe the distribution of aromatase in the hippocampal CA1 region of sham and IR 7d animals with or without RIP intervention. The results showed that in the sham and sham + RIP groups, aromatase was predominantly expressed in the cytoplasm of NeuN-positive cells. However, in IR 7d animals, there was strong co-localization of aromatase with GFAP. Interestingly, RIP intervention resulted in aromatase reemerging in NeuN-positive cells at IR 7d (Fig. 1B–C).

Moreover, the effect of RIP on BDE2 in the hippocampal CA1 region was examined after GCI. The immunofluorescence staining results showed (Fig. 1D, d1) that there was no significant change in E2 immunofluorescence intensity in the Sham + RIP group compared to the sham group. However, E2 immunofluorescence intensity was significantly reduced in the IR 7d group compared to the sham group. The use of letrozole, an aromatase activity inhibitor, significantly inhibited the upregulation of E2 induced by RIP. Meanwhile, E2 immunofluorescence intensity was significantly increased after RIP intervention.

### Effects of RIP Intervention on Astrocytic Phenotypes in the CA1 Region of the Hippocampus of IR 7d Rats

Recent studies have proposed that reactive astrocytes can be classified into two main types, A1 and A2. A1-astrocytes are induced by cytokines released from activated microglia, leading to aggravation of inflammatory impairments in response to ischemic insult. Conversely, astrocyte-derived estrogen can result in upregulation of A2-astrocytes, which exert neuroprotective roles [36, 9]. Given that RIP intervention altered protein expression and distribution of aromatase and BDE2 levels in IR 7d animals, the study investigated whether RIP could regulate the transformation of A1-A2, which might becontribute to neuroprotection and repair after GCI.

**Fig. 2:**
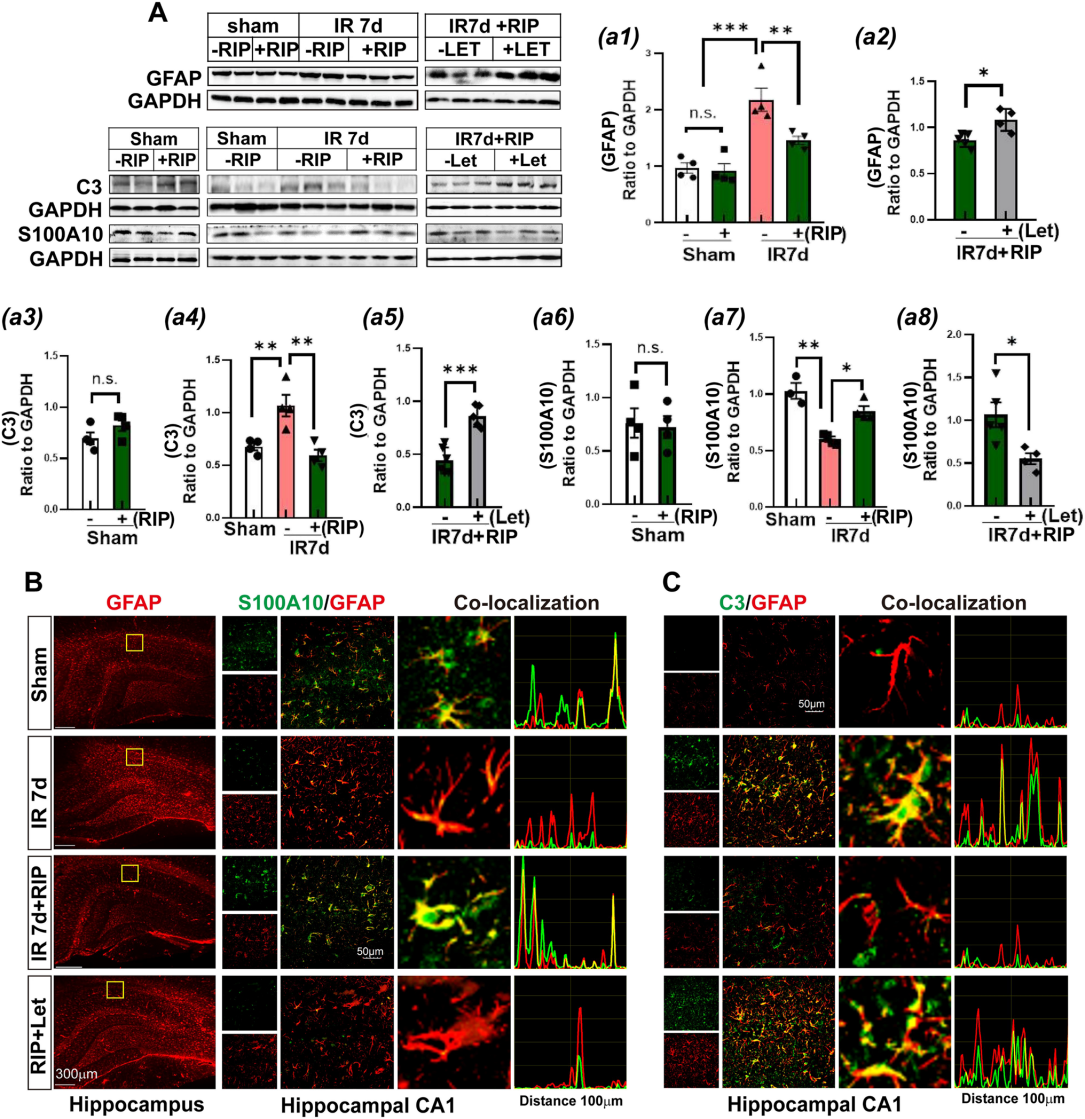
RIP reversed A1-A2 astrocyte polarization in the hippocampal CA1 region of OVX rats at 7d after GCI. **A-**a1**-**a8 Western blot analysis showed the protein expression of astrocyte PAN marker GFAP, A1-astrocyte marker C3, and A2-marker S100A10. Values are means ± SEM of determinations from each group. *P<0.05, **P<0.01, ***P<0.001, n.s. means no significance, *n*=3-5. GAPHD was used as the loading control. **B** The first column shows representative images of the whole hippocampus for GFAP immunofluorescence staining (red). The middle column showed double immunofluorescence staining of S100A10 (green) with GFAP (red), and the third column showed co-localization by fitting analysis of S100A10 with GFAP in the hippocampal CA1 region. **C** Representative images for C3 (green) and GFAP (red) and fitting analysis of their co-localization by fuji software. Magnification 10 × and scale bar 300 μm in the first column of Figure C; magnification ×40 and scale bar 50 μm in the second column of Figure B, and the first column of Figure C

GFAP (the hallmark of reactive astrocytes), S100A10 (a marker of A2-astrocytes), and C3 (a marker of A1-astrocytes) were detected in the hippocampal CA1 region. Western blot analysis showed that protein expression of GFAP and C3 significantly increased in the IR 7d group compared to sham animals, whereas S100A10 level decreased (Fig. 2A, a1–a8). Compared to the IR 7d group, RIP intervention significantly attenuated GFAP and C3 levels and enhanced S100A10 expression. Notably, treatment with letrozole profoundly reversed the effects of RIP. There was no significant change in protein expression of GFAP, S100A10, and C3 between sham and sham + RIP groups.

Double immunofluorescence staining of GFAP (red) with S100A10 (green) (Fig. 2B) and GFAP (red) with C3 (green) (Fig. 2C) demonstrated that RIP intervention significantly decreased the immunofluorescence intensity of C3 but elevated S100A10 levels compared to the IR 7d group. Furthermore, the fitting images displayed high co-localization of GFAP- and C3-positive staining in the IR 7d group and of GFAP- and S100A10-positive staining in the RIP-intervened animals, respectively. Letrozole treatment reversed the effects of RIP, showing a marked C3 expression increase and strong co-localization with GFAP, as well as a significant S100A10 expression decrease, which perfectly mirrored western blot results. Together, the results suggest that aromatase-BDE2 signaling induced by RIP intervention might exert neuroprotective roles by transforming A1-astrocytes to A2-astrocytes.

### Aromatase-E2 Signaling Induced by RIP Intervention Protects the Hippocampal CA1 Neurons and Improves the Spatial Memory Capacity of IR 7d Rats

**Fig. 3:**
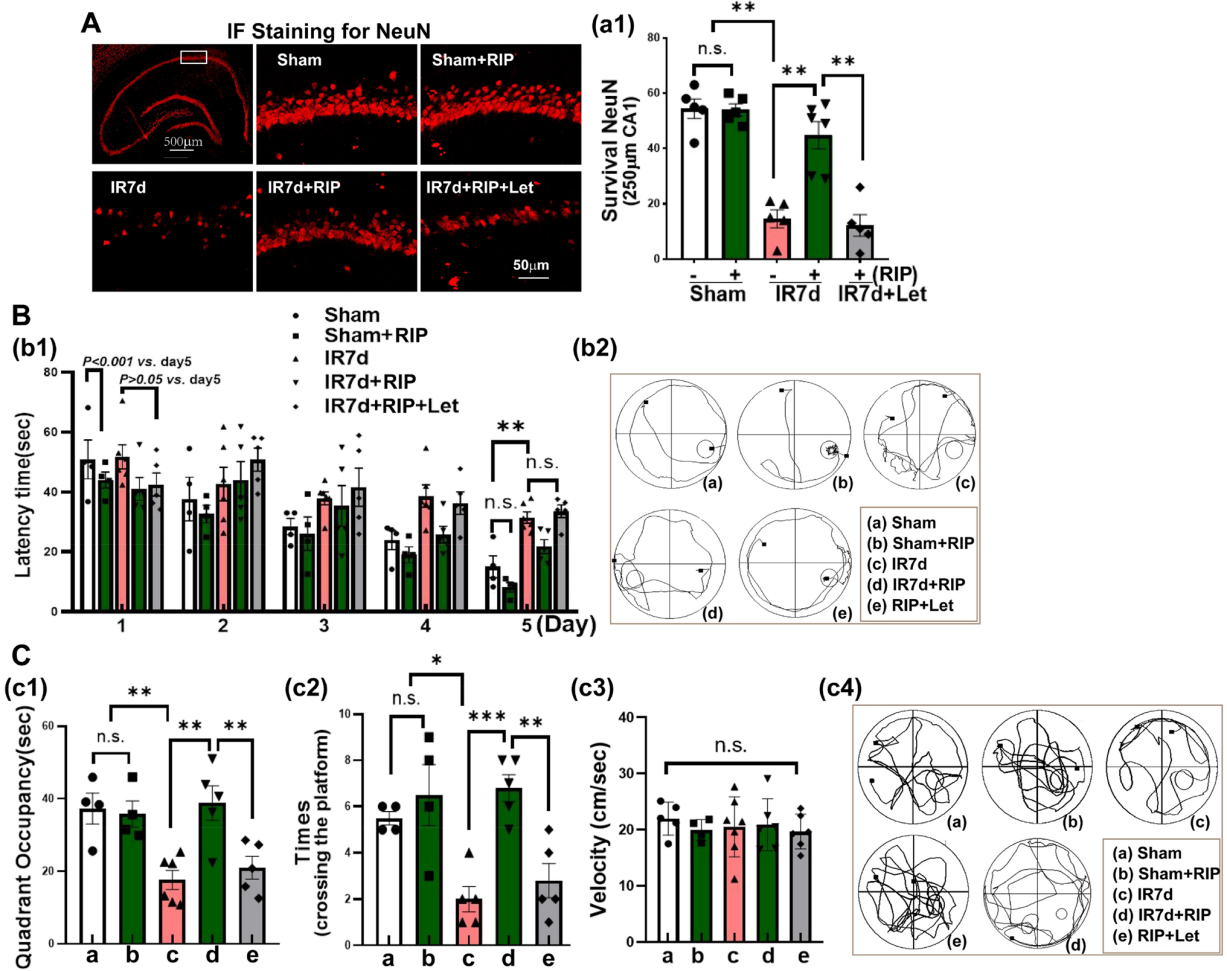
Aromatase inhibition abolished the neuroprotective role and cognitive improvement induced by RIP in OVX GCI rats. **A** The first image showed the whole hippocampus by immunofluorescent staining (IF) of survival neuron marker NeuN, and the rectangular indicates the position of the hippocampal CA1 region that was presented representative images of NeuN IF (red). a1 Quantitative analysis of the number of NeuN-positive cells per 250 μm length of medial CA1 pyramidal cell layer. Latency trial (**B**) and probe trial (**C**) results in the Moris water maze. b1 Time (sec) spent finding the submerged platform at reperfusion 4–7d after GCI (day 1–day 3 of MWM). c1 Exploration time spent in the quadrant that initially contained the platform on day 7 of MWM. c2 Times of the rats crossed the platform in the probe trial. The velocity of all group rats was shown in c3. Representative traces indicating the sample paths of the rats from the maze latency trials (b2) and the swimming traces from probe trials (c4). Data is expressed as mean ± SEM from four to six different animals (*n*=4-6). ^*^P<0.05, **P<0.01, ***P<0.001, n.s. means no significance. IR: Ischemia-reperfusion; RIP: Remote ischemic postconditioning; Let: letrozole.

In Fig. 3A, immunofluorescence staining was performed on NeuN (red), which is a marker for surviving neurons. The results showed that compared to the sham group, IR 7d animals had a significant reduction in the number of NeuN-positive cells in the hippocampal CA1 region. However, RIP intervention prevented this impairment and showed a significant increase in NeuN-positive cells. Interestingly, the protective effect of RIP on ischemic neurons was abolished by letrozole treatment in the hippocampal CA1 region.

To evaluate the cognitive function of rats, we conducted the Morris water maze test on the fourth day after GCI. During the 3-day training phase, rats in the Sham and Sham + RIP groups gradually reduced their time to locate the underwater platform. The rats of IR and RIP intervention did not show significant differences in finding the platform during the first 2 days, but by the third day, they were able to locate the platform in less time compared to the first day. However, rats treated with Letrozole showed no significant difference in their ability to find the platform during the 3-day latency trial. On the last day of the latency trial, the rats of IR 7d, RIP and letrozole groups displayed a significant increase in latency time to find the platform compared to the Sham and Sham + RIP groups. Nevertheless, there was no significant difference in the latency time to find the platform among the IR 7d, RIP, and letrozole-treated groups (as depicted in Fig. 3B–b1). Importantly, as observed during the probe trial, the rats belonging to the sham, sham + RIP, and IR 7d + RIP groups showed significantly better reference memory performance compared to the IR 7d rats, as evidenced by their increased time spent in the platform quadrant and the number of times they crossed the target platform. Interestingly, the cognitive protection effect was nullified upon administration of letrozole, as it resulted in reduced time spent in the platform quadrant and fewer platform crossings when compared to the RIP group. The velocity of all group rats exhibited no significance (Fig. 3C–c3). The rats’ swimming patterns during the latency and probe trials were visualized through the tracings presented in Fig. 3–b2 and Fig. 3–c4, respectively.

### Aromatase Inhibition Suppresses Pro-survival Signaling Induced by RIP in the Hippocampal CA1 Region at IR 7d

**Fig. 4:**
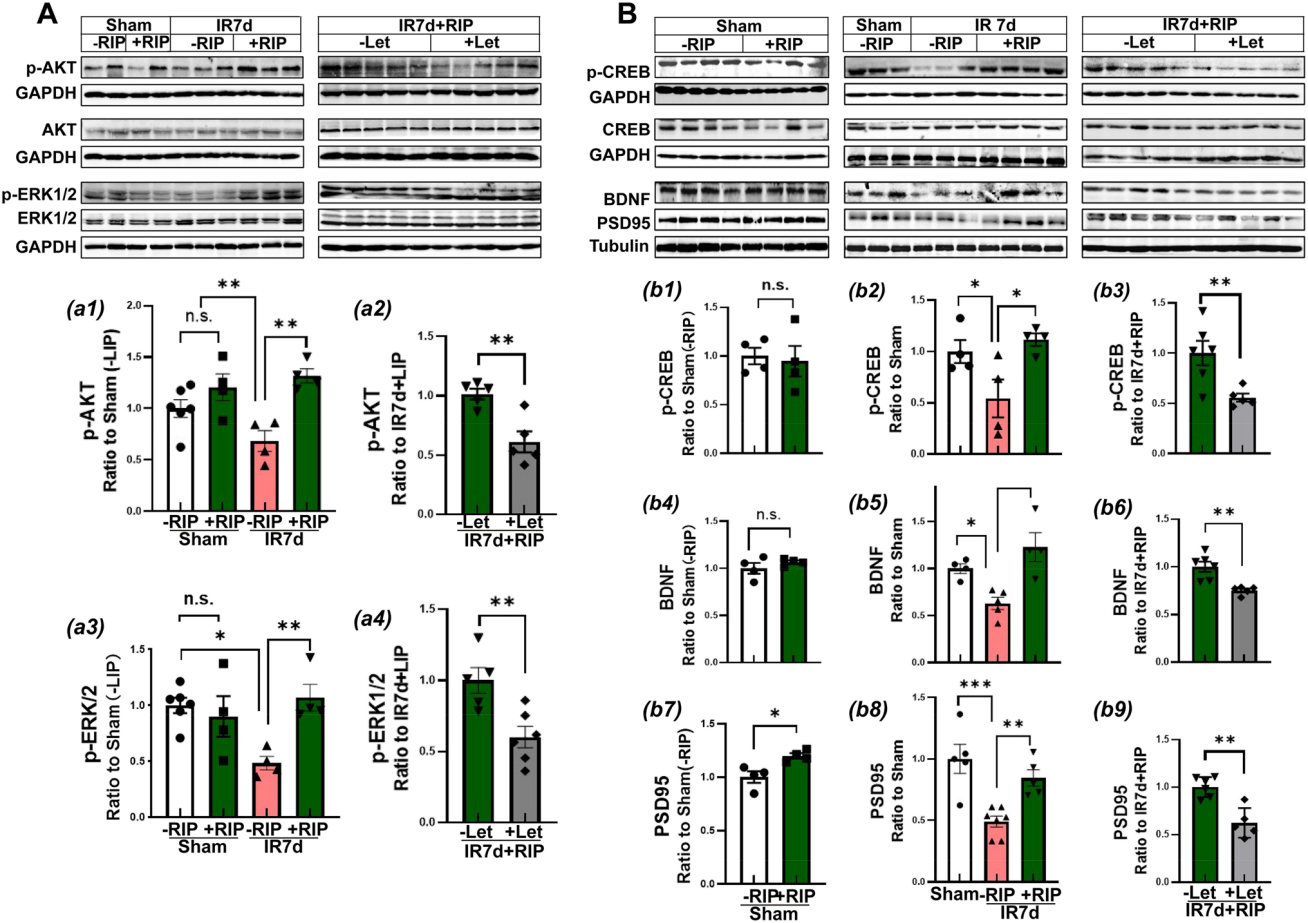
Aromatase inhibition suppressed pro-survival signaling induced by RIP in the hippocampal CA1 region of GCI rats. **A-**a1**-**a4 Western blot analysis showed phosphorylation (activation) of survival kinases AKT and ERK1/2 and their total protein expression. **B-**b1**-**b9 Western blot analysis showed the activation of transcription activator CREB (a well-known down-substrate of AKT or ERK1/2), brain-derived neurotrophic factor BDNF, and postsynaptic density protein PSD95. GAPHD or Tubulin was used as the loading control. Values are means ± SEM of determinations from each group. ^*^P<0.05, ^**^P<0.01, ^***^P<0.001, n.s. means no significance, *n*=4-6

We next investigated the activation of pro-survival signaling pathways AKT, ERK1/2, and CREB, as well as cognitive-related proteins BDNF and PSD95 in the hippocampal CA1 region at IR 7d. Western blot analysis revealed that IR 7d had a negative impact on the phosphorylation (activation) of AKT, ERK1/2 **(a1–a4)**, and CREB **(b1–b3)** and protein levels of BDNF and PSD95 compared to sham animals **(b4–b9)**. However, RIP intervention was able to reverse these changes and increased p-AKT, p-ERK1/2, p-CREB, BDNF, and PSD95 compared to the IR 7d group (Fig. 4A and B). Interesting, letrozole-administration was found to suppress the upregulation of these proteins and abolish the neuroprotective effects of RIP on ischemic rats. Despite these findings, the exact mechanism remains unclear and requires further investigation.

### RIP Intervention Suppressed Ca_V_1.2-CaMKIIα Signaling that Was Abolished by Aromatase Inhibition in the Hippocampal CA1 Region of IR7d Rats

**Fig. 5:**
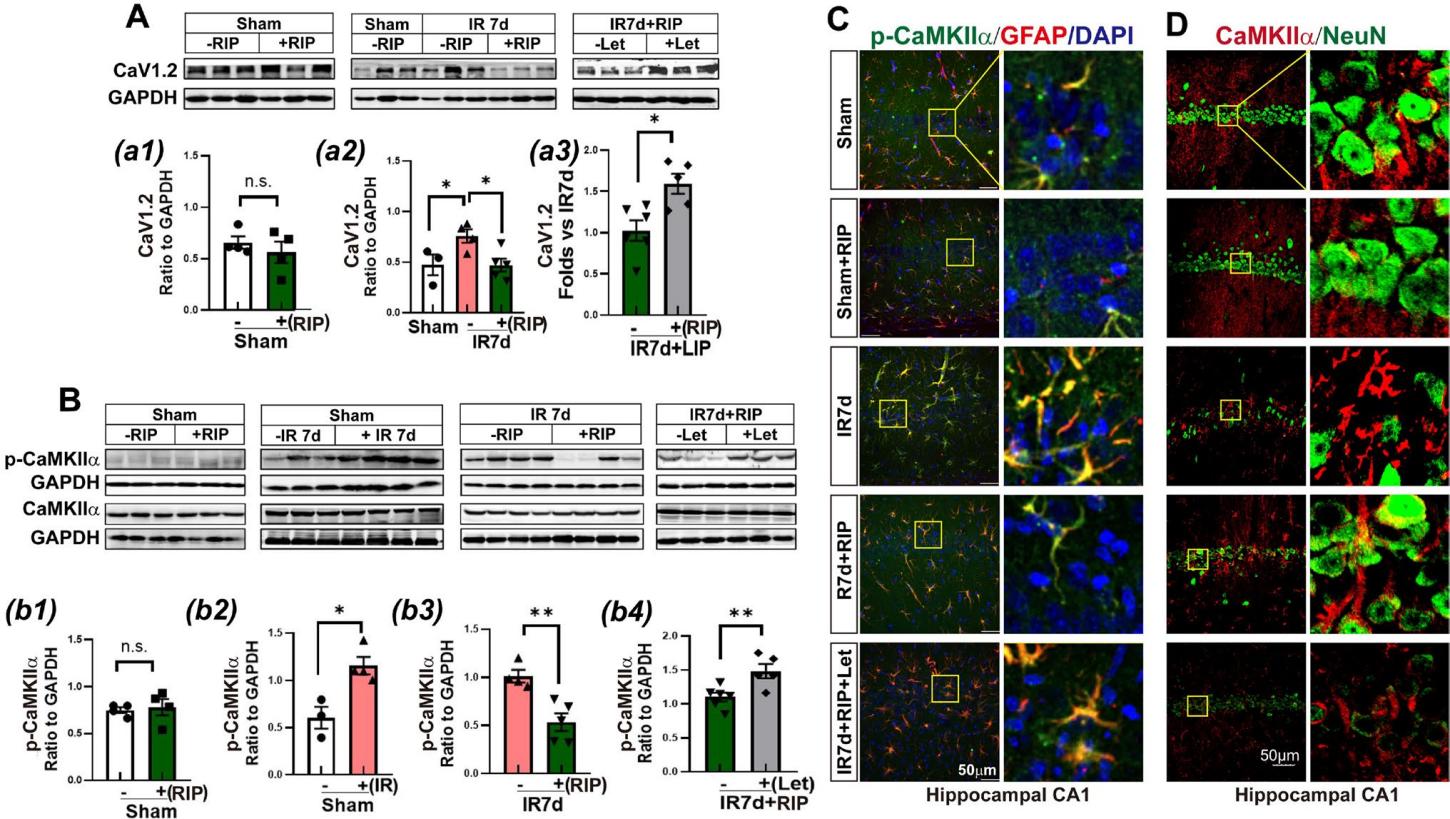
Letrozole abolished the effects of RIP on CaV1.2-CaMKIIα signaling in the hippocampal CA1 region of GCI 7d rats. Western blot analysis showed protein expression of CaV1.2 **A-**a1**-**a3, and CaMKIIα and p-CaMKIIα **B-**b1**-**b4 in the hippocampal CA1 region of indicated group animals. Values represent means ± SEM of determinations from each group. ^*^P<0.05, ^**^P<0.01, n.s. means no significance, *n*=3-6. GAPHD was a loading control. Double immunofluorescence staining for p-CaMKIIα (green) with GFAP (red) **C**, or CaMKIIα (red) with GFAP (green) **D**, and showing co-localization distribution of p-CaMKIIα or CaMKIIα with GFAP (yellow) in the indicated groups. Magnification ×40 and scale bar 50 μm

Neuronal injury after cerebral ischemia is frequently associated with calcium overload. Research has shown that the CaV1.2 calcium channel, which is a major member of L-VGCC in the central system, is responsible for depolarization-induced Ca^2+^ influx that contributes to ischemic neuronal injury and is mediated by astrocytes [37]. Furthermore, research suggests that estrogen treatment can promote cognitive function by increasing the degradation of CaV1.2 in the brain through ubiquitination [38]. CaMKIIα, an enzyme that is highly sensitive to intracellular Ca^2+^ concentration, can undergo upregulation in response to cerebral ischemic insult. Increased calcium level can upregulate CaMKIIα phosphorylation, leading to the adverse phosphorylation of the CaV1.2 channel and the enhancement of Ca^2+^ influx [39]. However, it has been found that p-CaMKIIα can lead to phosphorylation (inactivation) of aromatase, thuspreventing estrogen biosynthesis [40, 41]. Therefore, we next asked whether Ca_V_1.2-CaMKIIα signaling was involved in BDE2 biosynthesis with or without RIP intervention. As shown in Fig. 5A, B, western blot analysis revealed that IR 7d significantly upregulated Ca_V_1.2 protein expression (Fig. 5A, a2) and p-CaMKIIα (Fig. 5B, b2) in the hippocampal CA1 region compared with the Sham group. However, RIP intervention prevented the increases, resulting in lower levels of CaV1.2 expression (Fig. 5A, a2) and CaMKIIα phosphorylation (Fig. 5B, b3) than those in the IR 7d group. Interestingly, letrozole-administration suppressed the effects of RIP, as evidenced by increased CaV1.2 and p-CaMKIIα levels, compared to RIP animals (Fig. 5A–a3, B–b4). There was no significant difference in CaMKIIα expression among the groups (Fig. 5B).

Furthermore, we investigated activation (p-CaMKIIα), protein expression (CaMKIIα), and cell-type distribution of CaMKIIα by double immunofluorescence staining in the hippocampal CA1 region. The results showed that fluorescence intensity of p-CaMKIIα (green) was significantly increased in IR 7d and letrozole-treated groups mainly distributed in astrocyte marker GFAP-positive cells (red) compared to sham, or RIP intervented animals (Fig. 5C). Moreover, the fluorescence distribution of CaMKIIα (red) also occurred alternation, showing higher expression in neuronal marker NeuN-positive cells (green) in sham and RIP groups and higher expression in glial cells in IR 7d or letrozole-treated animals (Fig. 5D). The fluorescence distribution of CaMKIIα also showed a similar pattern with aromatase, indicating that CaV1.2-CaMKIIα signaling might play a role in BDE2 biosynthesis, potentially involving the mediation of astrocytes (Fig. 5D).

### CaMKIIα Specific Inhibitor TatCN21 Peptide Suppressed Astrocyte Reactivity and Upregulated Aromatase-BDE2 Levels in the Hippocampal CA1 Region at IR 7d

**Fig. 6:**
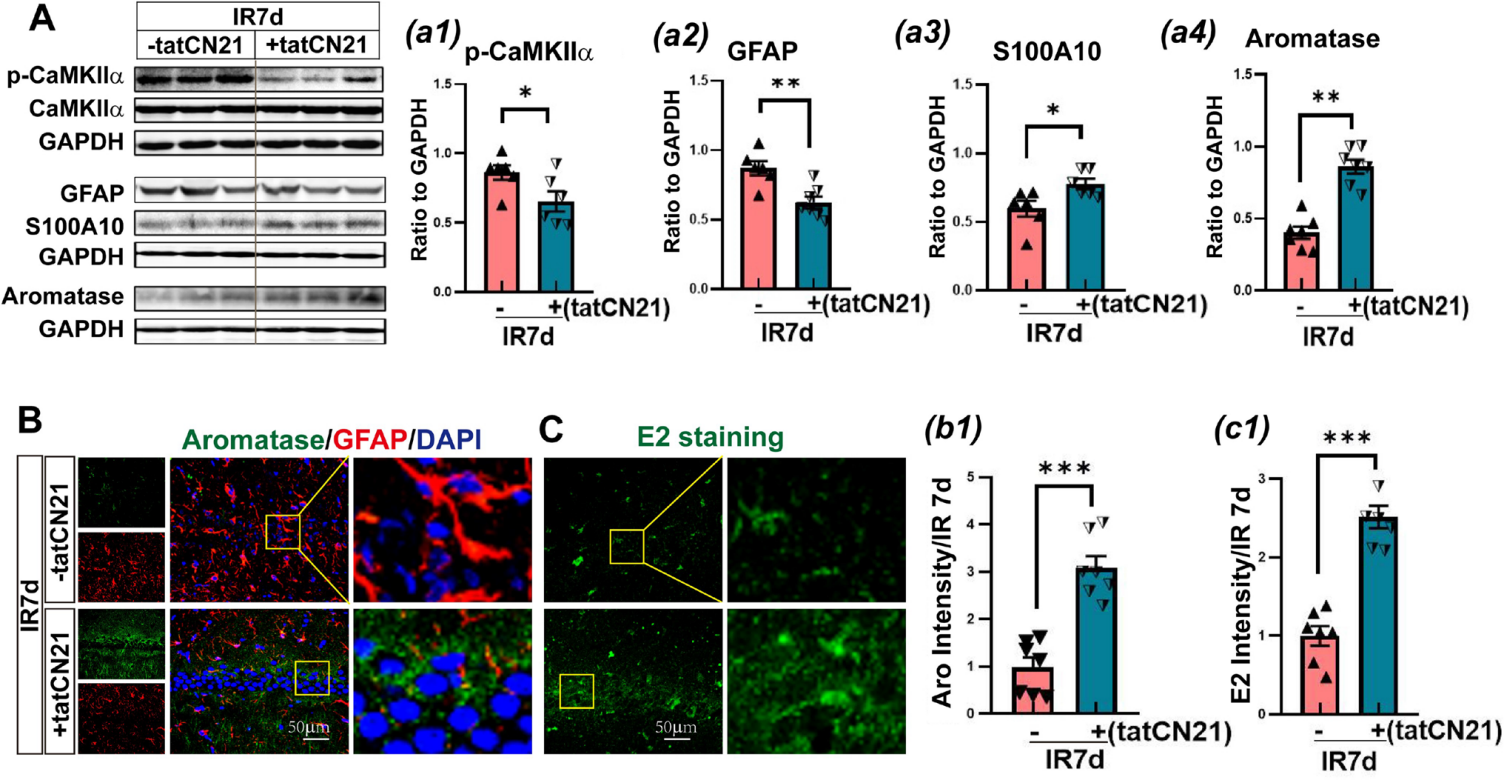
Inhibition of CaMKII α activity by tatCN21 modulated aromatase-BDE2 signaling in astrocytes in the hippocampal CA1 region of GCI 7d rats. **A-**a1**-**a4 Western blot analysis showed the effect of tatCN21 on the expression of astrocyte marker GFAP and S100A10 proteins and aromatase. The values were presented as means ± SEM and were determined from each group. ^*^P<0.05, ^**^P<0.01, ^***^P<0.001, n.s. means no significance, *n*=6–7. GAPHD was used as the loading control. **B**–b1 Immunofluorescence staining for aromatase (green), GFAP (red), and DAPI (blue) in the hippocampal CA1 region in the indicated groups. **C**–c1 Immunofluorescence staining for E2 (green) in the hippocampal CA1 region in the indicated groups. Magnification ×40 and scale bar 50 μm in the third column of Figure B, and the second column of Figure C

We first detected CaMKIIα activation and protein expression by TatCN21 peptide, a specific inhibitor of CaMKIIα activity, at IR 7d in the hippocampal CA1 region. The western blot analysis revealed that p-CaMKIIα level was significantly decreased after treatment with tatCN21 compared to the IR 7d group, while CaMKIIα protein expression had no significant change, indicating successful intervention of tatCN21 (Fig. 6A–a1).

We subsequently observed the effects of tatCN21 on astrocyte reactivity and aromatase protein expression by western blotting analysis. As shown in Fig. 6A, a2–a4, compared with the IR 7d group, tatCN21 treatment significantly reduced GFAP protein expression in the hippocampal CA1 region; however, tatCN21 markedly elevated protein expression of S100A10 and aromatase. Immunofluorescence staining also showed a strong fluorescence intensity of aromatase (green), but weak fluorescence intensity of GFAP (red) in tatCN21-treated animals compared to IR 7d group (Fig. 6B, b1). The effects of tatCN21 treatment on astrocyte reactivity and aromatase-BDE2 signaling are similar to RIP intervention (Fig. 6C, c1). DAPI (blue) was counter-stained nuclei.

### CaMKIIα Specific Inhibitor TatCN21 Peptide Exerted Neuroprotective Effects on Ischemic Hippocampal Neurons After GCI

**Fig. 7:**
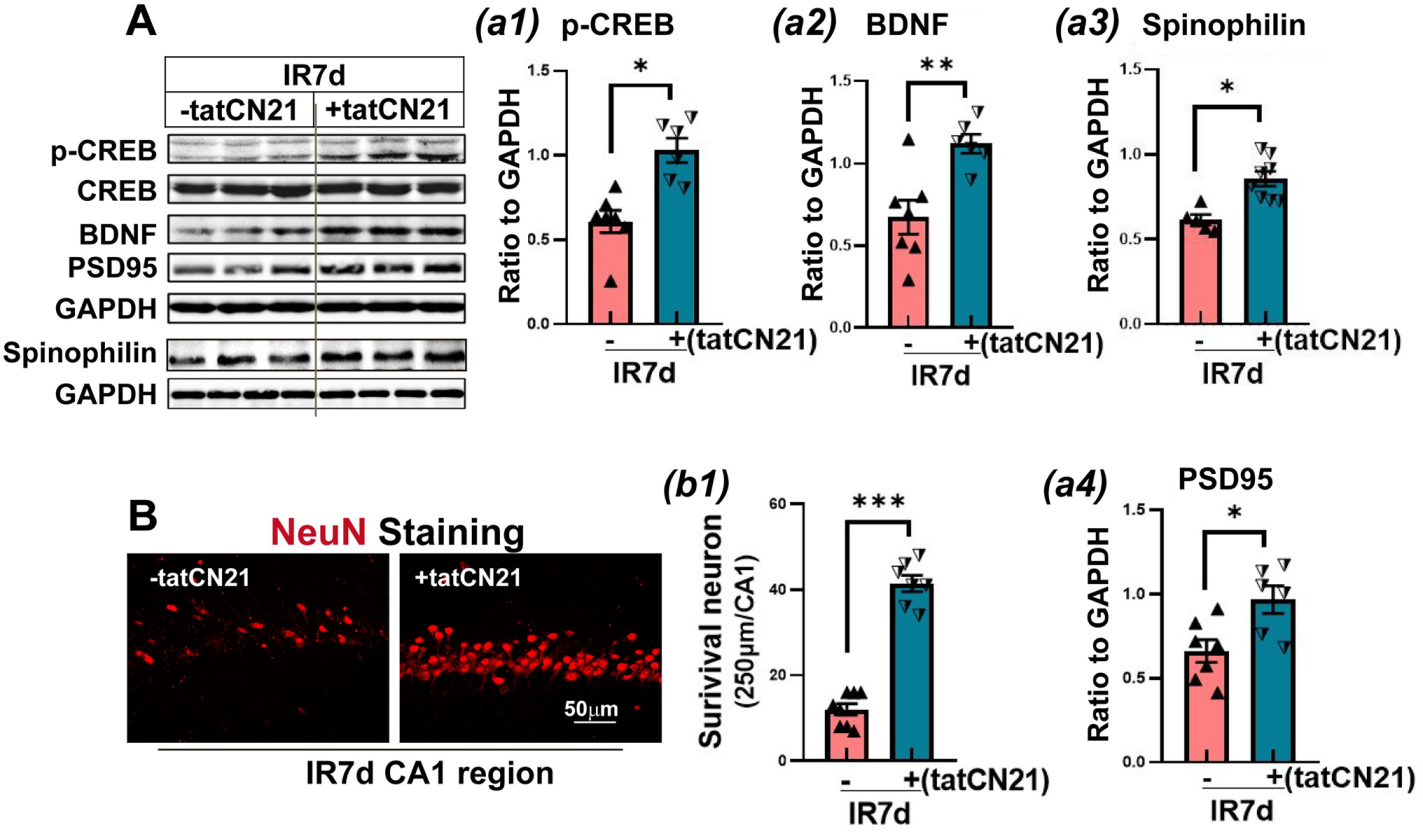
Inhibition of CaMKII α activity by tatCN21 protected neurons in the hippocampal CA1 region of GCI 7d rats. **A-**a1**-**a4 Western blot analysis showed the protein expression of survival-promoting protein transcription activator CREB/p- CREB, brain-derived neurotrophic factor BDNF, spinophilin, and postsynaptic density protein PSD95. Values are means ± SEM of determinations from each group. ^*^P<0.05, ^**^P<0.01, ^***^P<0.001, n.s. means no significance, *n*=6-7. GAPHD was used as the loading control. **B**–b1 Representative images of NeuN immunofluorescence staining (red) in the hippocampal CA1 region in the indicated groups. Quantitative analysis of the number of NeuN-positive cells per 250 μm length of medial CA1 pyramidal cell layer. Magnification ×40 and scale bar 50 μm

Finally, we examined the protective effect of tatCN21 on ischemic hippocampal neurons. Western blot analysis showed that tatCN21 not only upregulated the protein expression of p-CREB and BDNF in the hippocampal CA1 region but also significantly enhanced the protein expression of synaptic proteins spinophilin and PSD95 compared to IR 7d group (Fig. 7A, a1–a4). There was no significant change in CREB protein level. Furthermore, immunofluorescence staining results showed that tatCN21 significantly increased the number of surviving neuronal marker NeuN (red) in the hippocampal CA1 region compared to the IR 7d group (Fig. 7B, b1).

## Discussion

Based on the results of our study, we have proposed the hypothesis that suggests a causal relationship between RIP and aromatase-BDE2 signaling. This assumption is based on the following facts: Firstly, both RIP and BDE2 are endogenous neuro-protective properties. RIP is known to produce endogenous protective mechanisms, such as NO, adenosine, and bradykinin, which increase tolerance to severe ischemic events [42]. This suggests that there may be a link between RIP and the production of endogenous protective mechanisms, such as BDE2 signaling, which could contribute to the observed neuroprotective effects of RIP.This resistance is useful in delaying injury after cerebral ischemia or traumatic brain injury [43–46]. Secondly, astrocytes are the common important targets of their regulation and action [29]. RIP can reduce astrocyte reactivity in the MCAO rat model, protecting ischemic neurons by regulating aquaporin in astrocytes [28]. Furthermore, ischemic cerebral insult and traumatic brain injury not only activate astrocytes but also upregulate aromatase activity [45, 47]. Endogenous E2 produced by astrocytes contributes to neurogenesis and synapse formation [48] and promotes the processing of neurodegenerative diseases [49]. Gatson et al. reveal that in primary cortex astrocyte culture, 10-40 mmHg pressure can upregulate the expression and activity of aromatase and increase E2 level [47].

To prove the hypothesis, we examined the time course of aromatase protein in the hippocampal CA1 region after GCI. Our findings showed that aromatase protein had no significant change in the sham and IR 3h groups, with or without RIP, and there was no statistical difference between the groups. However, a significant increase in aromatase protein was observed at IR 3d compared to sham animals. Furthermore, at IR 7d, the aromatase protein and E2 levels sharply decreased compared to sham animals, indicating insufficient resistance. We suggest that the aromatase-BDE2 signal resists the secondary injury after ischemia at IR 3d, but eventually leads to the loss of hippocampal neurons and cognitive impairment at IR 7d due to insufficient resistance. In previous research, we found that protein expression and activity of aromatase were significantly increased at IR 3d, and letrozole administration worsened hippocampal neuronal damage [16].

Notably, RIP not only increased the expression of aromatase and E2 levels but also activated Akt and CREB pro-survival signal pathways while elevating the expression of the synaptic proteins in the hippocampal CA1 region. This relusted in animprovement in the memory function of IR 7d rats. Brain injury can lead to ion imbalance and CREB activation, ultimately increasing aromatase protein expression [50, 51]. Immunofluorescence staining for aromatase with astrocyte marker GFAP or survival neuron marker NeuN revealed that aromatase was highly expressed in survival neurons and reactive astrocytes. The findings suggest that RIP may paly a role in mediating astrocyte reactivity through an aromatase-BDE2-dependent pathway, ultimately promoting the repair of ischemic neurons.

Recently, researchers have identified two subtypes of reactive astrocytes, A1 and A2, based on their functions. A1 astrocytes are known to secrete neurotoxins, leading to the rapid death of neurons and oligodendrocytes, while A2 astrocytes promote neuronal survival and repair after brain injury [29, 52]. In the current study, RIP intervention was found todecrease GFAP protein, and upregulate S100A10, a marker of A2-astrocytes, while downregulating C3, a marker of A1-astrocytes in the hippocampal CA1 region of IR 7d rats. Letrozole administration abolished the mediation of reactive astrocytes by RIP, as evidence of strong astrogliosis, decreased S100A10, and increased C3 in the hippocampal CA1 of IR 7d. Previous studies have reported that RIP promotes brain recovery in a mouse model of ischemic stroke by regulating the plasticity of reactive astrocytes [28]. Consistent with these results, another study by Brann et al. demonstrated that astrocyte-derived E2 can regulate reactive astrogliosis and is neuroprotective following ischemic brain injury by aromatase-knock-out mice, specifically in fore-brain astrocytes [9]. Our findings and those of others further confirm that RIP exerts protective effects through the regulation of reactive astrogliosis, involving endogenous aromatase-E2 signaling. However, the possible mechanism needs further exploration.

Calcium overload is a well-known mechanism of cerebral ischemia-reperfusion injury and is also a core mechanism of many other processes such as glutamate excitotoxicity, oxidative stress, and inflammation [53]. The L-type voltage-gated calcium channels (L-VGCC) are the primary channels responsible for calcium influx and participate in the regulation of calcium homeostasis, synaptic plasticity, gene expression, and hormone secretion [54–57]. The L-VGCC family includes four members of the Cav1 family (Cav1.1, Cav1.2, Cav1.3, Cav1.4), with Cav1.2 being the main subtype of L-VGCC, accounting for about 87% [58, 59]. Abnormal expression of Cav1.2 molecules can affect extracellular calcium influx, as observed in vitro. Conditional knockdown of Cav1.2 in astrocytes can block calcium influx by 80% and reduce LPS-induced activation and proliferation of astrocytes [60]. Cav1.2 α Subunit deletion in mouse corpus callosum astrocytes can reduce neuroinflammation and promote remyelination [37]. In a rat model, three weeks of chronic restraint stress to induce depression significantly increased Cav1.2 and its downstream CaM-NFAT signaling in the hippocampus [61]. Another downstream substrate of the Cav1.2-CaM signaling pathway is CaMKIIα, the most sensitive protein kinase to cellular calcium concentration fluctuation. When excessive calcium influx is stimulated by brain injury, CaMKIIα is automatically phosphorylated at Thr286, resulting in phosphorylation of its downstream substrate or initiation of transcription factors, such as N-methyl-D-aspartate receptor subunit 2B (NR2B), protein kinase C (PKC) [62–64]. Additionally, p-CaMKIIα can provide feedback to upregulate Cav1.2 phosphorylation, resulting in increased activity of the Cav1.2 channel [39]. It is reported that calcium influx increased 4-6 times after phosphorylation of Cav1.2 [65, 66]. In this context, the effects of RIP on Cav1.2-CaMKIIα signaling in the hippocampal CA1 region of IR 7d rats were investigated. It was found that CaV1.2 protein expression and the ratio of p-CaMKIIα/CaMKIIα significantly enhanced at IR 7d compared to the sham group, while RIP markedly suppressed the enhancement.

The study found that Immunofluorescence staining showed that both Cav1.2 and p-CaMKII displayed a similar pattern as aromatase in cell type distribution; under basal conditions, both proteins were present in the cytoplasmic domain of neurons but strongly elevated in reactive astrocytes at IR 7d. However, RIP was able to reverse the distribution of these proteins. CaMKIIα is the most abundant CaMKII isoform in the brain in neurons, but there is limited information on CaMKIIα in other cell types within the neurovascular unit (such as endothelial cells, pericytes, smooth muscle cells, astrocytes, microglia, and extracellular matrix) [67, 68]. The distribution of p-CaMKIIα was verified by repeating the immunofluorescence staining using two different antibodies of p-CaMKIIα (No. cell signaling #3361, P1005-305) and CaMKIIα (Santa Cruz, sc5391; Cell signaling technology, #50049 and #3357S); both results showed that p-CaMKIIα/CaMKIIα was strongly colocalized with GFAP+ cells in IR 7d rats, and mainly presented in NeuN+ cells after RIP intervention but with lower expression compared to IR 7d group. CaMKIIα is known to have a double-edged sword effect, promoting both neuronal survival or death [69]. To further clarify the key role of CaMKIIα following GCI, tatCN21, a specific activity inhibitory peptide of CaMKIIα, was administered and found to reduce the level of p-CaMKIIα and protect hippocampal neurons against ischemic insult. Additionally, tatCN21upregulated protein expression of aromatase and S100A10, and the E2 level was significantly increased in the hippocampal CA1 region compared to IR 7d animals. These findings are consistent with previous evidence that has shown that hypoxic preconditioning reduces p-CaMKIIα in rats with transient global cerebral ischemia, ultimately offering protection to ischemic neurons [68, 70, 71].

## Conclusion

**Fig. 8:**
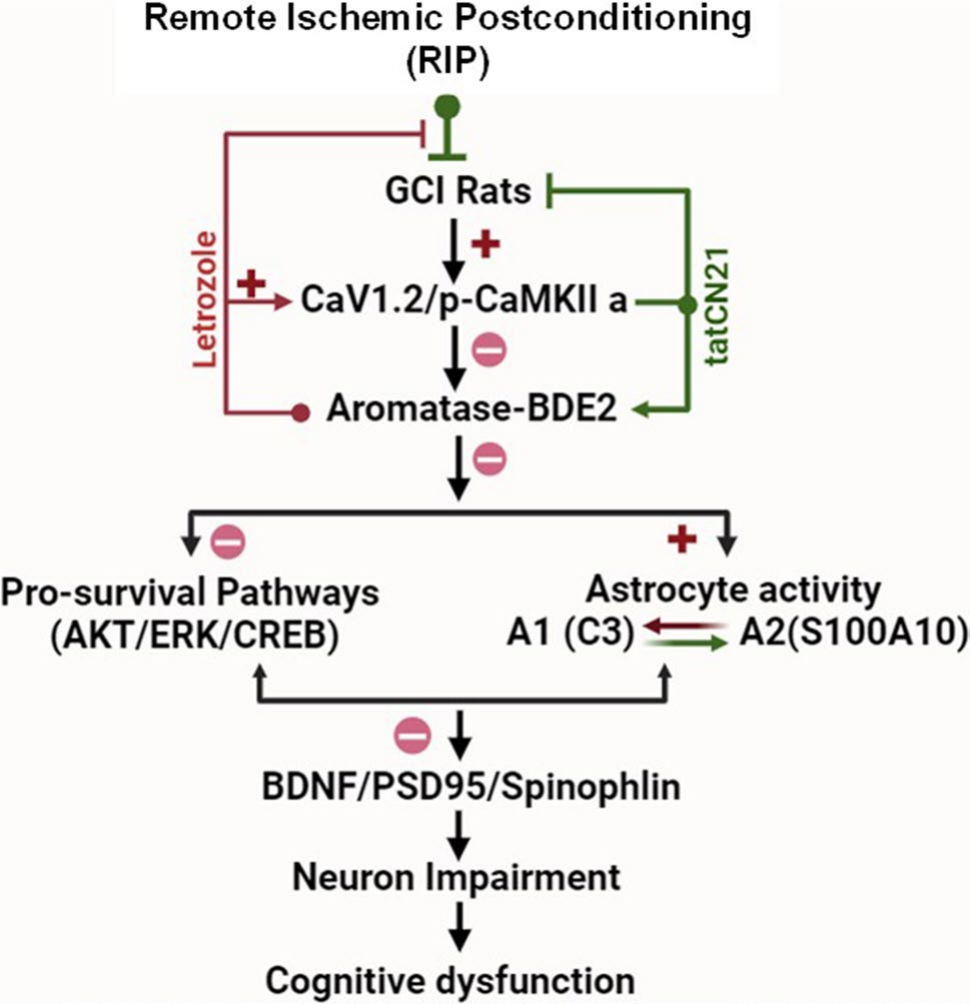
Schematic representation of the research strategy. Global cerebral ischemia (GCI) in rats can cause an increase in the CaV1.2-CaMKIIα signaling pathway in the hippocampus CAl region, resulting in the inhibition of BDE2 biosynthesis and downregulation of pro-survival pathways AKT, ERK1/2, and CREB, while also promoting A1-astrocyte activity. However, the performance of RIP after GCI significantly reverses these damages, increased protein levels of BDNF, synaptic protein PSD95, spinophlin, A2-astrocyte, and ultimately mitigated cognitive dysfunction. The research also utilized an aromatase specific inhibitor letrozole and an inhibitor peptide TatCN21 of CaMlKllα activity to confirm that RIP can induce aromatase-BDE2 through CaV1.2-CaMlKllα signaling and has beneficial effects on astrocytes

Our research findings, as illustrated in Fig. 8, reveal that RIP prevents neuronal impairment and promotes learning and memory function induced by GCI. Our study is significant becauseit is the first one to demonstrate this ability, which is attributed to the increased activation of Aromatase-BDE2 signaling. This mechanism effectively inhibits the CaV1.2-CaMKIIα calcium channel in the hippocampal CA1 region of OVX rats, thus offering a promising avenue for restoring hippocampal CA1 neurons and ultimately improving cognitive function in individuals who have undergone cardiac arrest.

## Data Availability

The raw data supporting the conclusions of this article will be made available by the authors, without undue reservation.
